# Supplementary material for: Synergistic regulation of Notch signaling by different O-glycans promotes hematopoiesis
Source: Front Immunol. 2023 Sep 19;14:1097332. doi: 10.3389/fimmu.2023.1097332 (PMC10546201; doi:10.3389/fimmu.2023.1097332)
Supplement: Supplementary file 1 [file DataSheet_1.pdf]

Supplementary information to

Synergistic regulation of Notch signaling by different O-glycans promotes hematopoiesis

Ankit Tanwar,[1] Pamela Stanley[1]

[1]Department of Cell Biology, Albert Einstein College of Medicine, New York, NY, USA

Short Title: O-glycans promote lymphopoiesis and myelopoiesis

Corresponding author:

Pamela Stanley

Department of Cell Biology,

Albert Einstein College of Medicine

New York, NY, 10461 USA

Email: pamela.stanley@einsteinmed.edu

Phone: 718-430-3346

Fax: 718-430-8574

Supplementary Appendix

**Supplementary Figures**	**Page**
**Supplementary Figure 1.** Gating strategy for BM hematopoietic cell subsets. Related to Figure 2, Figure 5 and Supplementary Figure 4	3
**Supplementary Figure 2.** Gating strategy for thymic immune cell subsets. Related to Figure 2, Figure 6, Supplementary Figures 4 and 5	4
**Supplementary Figure 3.** Gating strategy for splenic immune cell subsets. Related to Figure 2, Figure 7, Supplementary Figures 4 and 5	5
**Supplementary Figure 4.** Immune cell development in *Eogt*[+/+] versus *Eogt*[+/-] mice. Related to Figure 2	6
**Supplementary Figure 5.** Immune cell subsets from Control versus *Eogt*[-/-] mice. Related to Figure 2	7
**Supplementary Figure 6.** Gating strategy for BM hematopoietic cell subsets. Related to Figure 5	8
**Supplementary Figure 7.** Hematopoietic stem progenitors in control, Pof cKO and EPof dKO BM. Related to Figure 5	9
**Supplementary Figure 8.** Notch ligand binding to Lin-Sca1+ cells. Related to Figure 5	10
**Supplementary Figure 9.** Thymic immune cell subsets in Pof cKO and EPof dKO mice. Related to Figure 6	11
**Supplementary Figure 10.** Splenic immune cell subsets in Pof cKO and EPof dKO mice. Related to Figure 7	12
**Supplementary Tables**	
**Supplementary Table 1.** Primer sequences used in genotyping and qRT-PCR	13
**Supplementary Table 2.** Antibodies used in this work	14-15

Bone Marrow

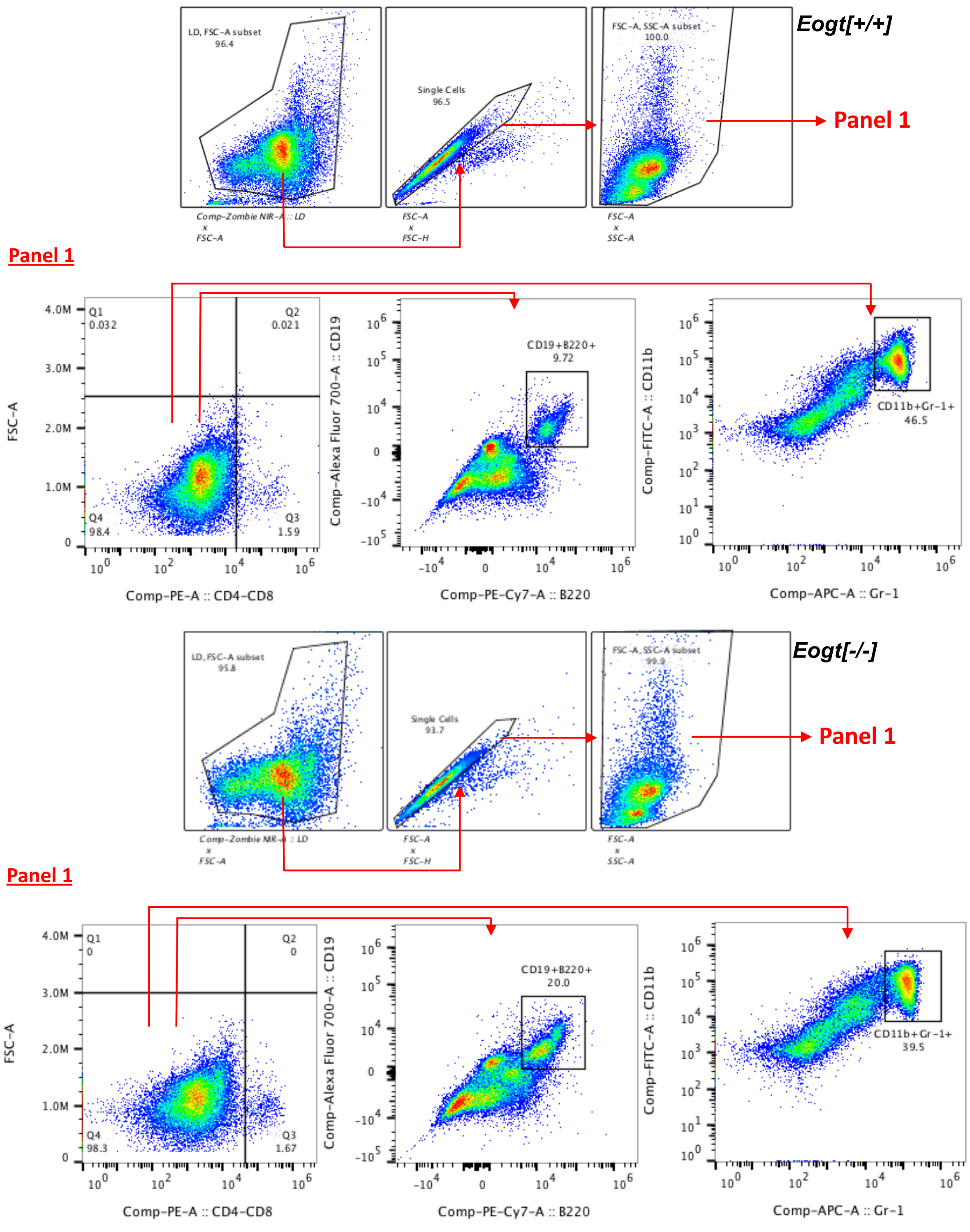

Supplementary Figure 1. Gating strategy for BM immune cell subsets. Related to Figure 2, Figure 5, and Supplementary Figure 4.

Flow cytometry profiles of BM cells from a 7-8 week *Eogt*[+/+] or *Eogt*[-/-] mouse selected after gating on live cells using Zombie NIR dye in the Cytek™ Aurora flow cytometer. Live singlets were selected (FSC-H vs FSC-A) and the main population (SSC-A vs FSC-A) was plotted for CD4+CD8+ vs FSC-A and gated on CD4-CD8- cells to compare CD19+ vs B220+ or CD11b+ vs Gr-1+ cell populations.

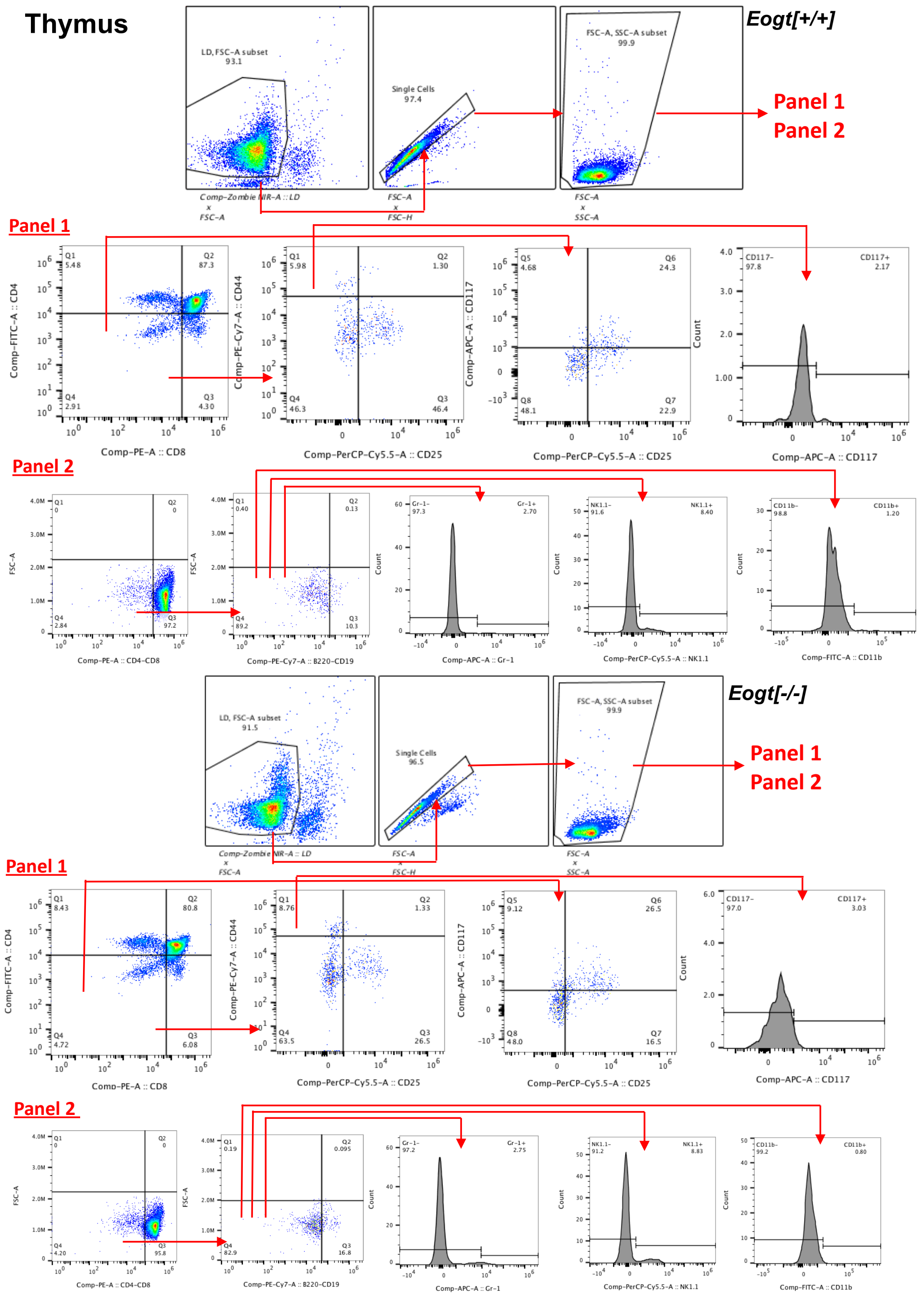

Supplementary Figure 2. Gating strategy for thymic immune cell subsets. Related to Figure 2, Figure 6 and Supplementary Figures 4 and 5.

Flow cytometry profiles of thymocytes from a 7-8 week *Eogt*[+/+] or *Eogt*[-/-] mouse selected after gating on live cells using Zombie NIR dye in the Cytek^{TM} Aurora flow cytometer. Live singlets were selected (FSC-H vs FSC-A) and the main population (SSC-A vs FSC-A) was plotted for Panel 1 antibodies to CD4 vs CD8 to determine frequencies and absolute numbers of CD4+ SP and CD8+ SP T cells, CD4+CD8+ DP T cells and CD4-CD8- DN T cells. The DN T cell population was plotted for CD44 vs CD25 to determine frequencies and absolute numbers of CD44+CD25- (DN1), CD44+CD25+ (DN2), CD44-CD25+ (DN3) and CD44-CD25- (DN4) T cell progenitors, and CD117+ DN1 cells (ETP). Panel 2 antibodies were used to plot FSC-A vs CD4/CD8 to gate on CD4-CD8- cells to then determine frequencies and absolute numbers of non-T cells in the thymus by plotting CD19 vs B220, and gating on CD19-B220- to determine non-B Gr-1+, NK1.1+, CD11b+ cells. Flow cytometry plots show B cells (B220+/CD19+), myeloid cells (CD11b+), granulocytes (Gr-1+), and natural killer T-cells (NK1.1+).

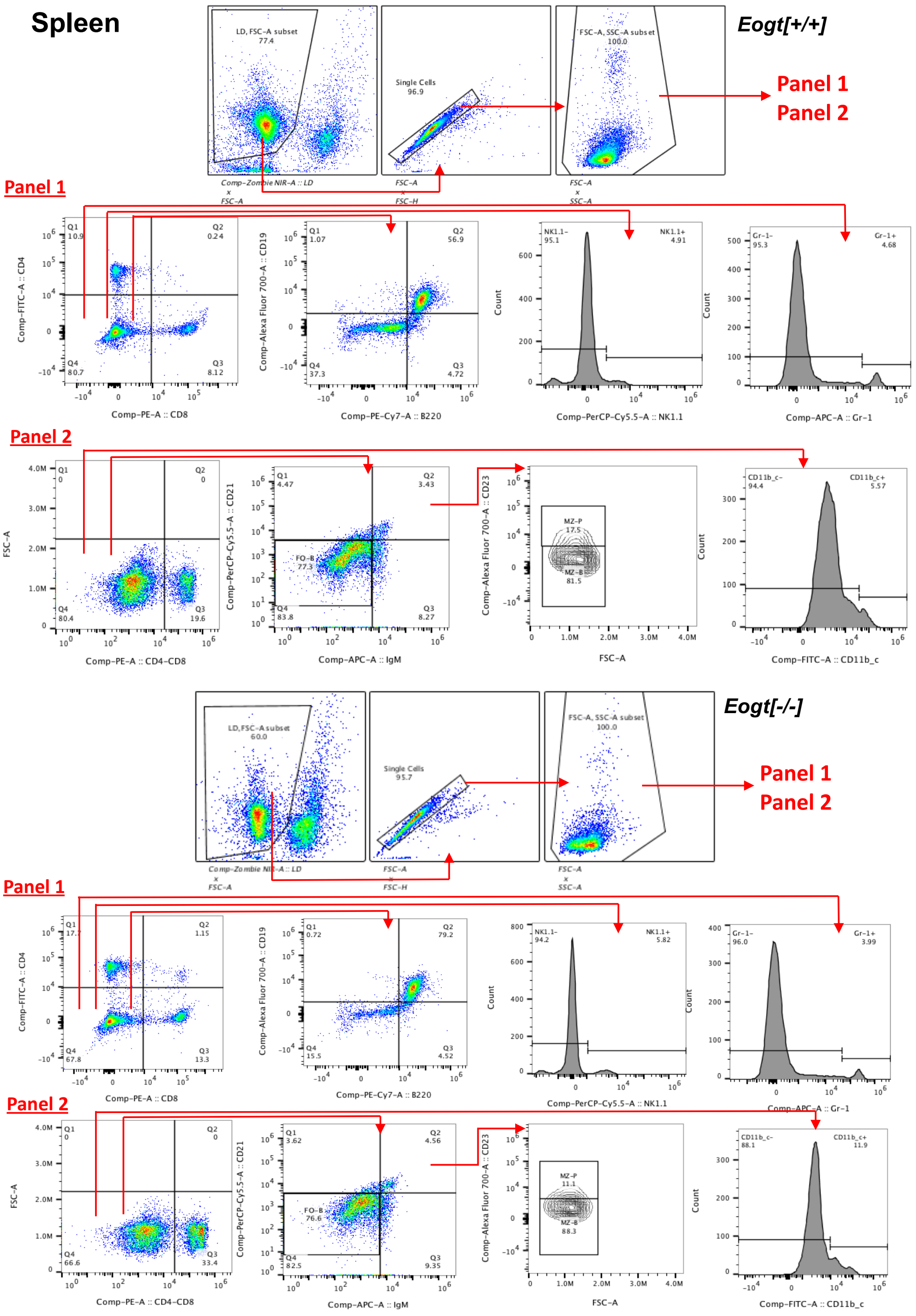

Supplementary Figure 3. Gating strategy for splenic immune cell subsets. Related to Figure 2, Figure 7 and Supplementary Figure 4 and 5.

Flow cytometry profiles of splenocytes from a 7-8 week *Eogt*[+/+] or *Eogt*[-/-] mouse selected after gating on live cells using Zombie NIR dye in the Cytek^{TM} Aurora flow cytometer. Live singlets were selected (FSC-H vs FSC-A) and the main population (SSC-A vs FSC-A) was used with Panel 1 antibodies to plot CD4 vs CD8**;** non-T cells (CD4-CD8-) were used to plot CD19 vs B220 to determine frequencies and absolute numbers of B cells, natural killer T cells (NK1.1), or granulocytes (Gr-1)**.** Panel 2 antibodies were used with the main population of singlets to plot FSC-A vs CD4/CD8**;** non-T cells (CD4-/CD8-) were used to plot CD21 vs IgM or CD11b/c**;** CD21+IgM+ were used to plot CD23+ vs FSC-A. Flow cytometric profiles show Fo-B cells (IgM $^{int/lo}$ CD21+) and histogram showing CD11b. The IgM+CD21+ subset was further subdivided into MZ-P and MZ-B cells based on forward scatter and CD23 expression.

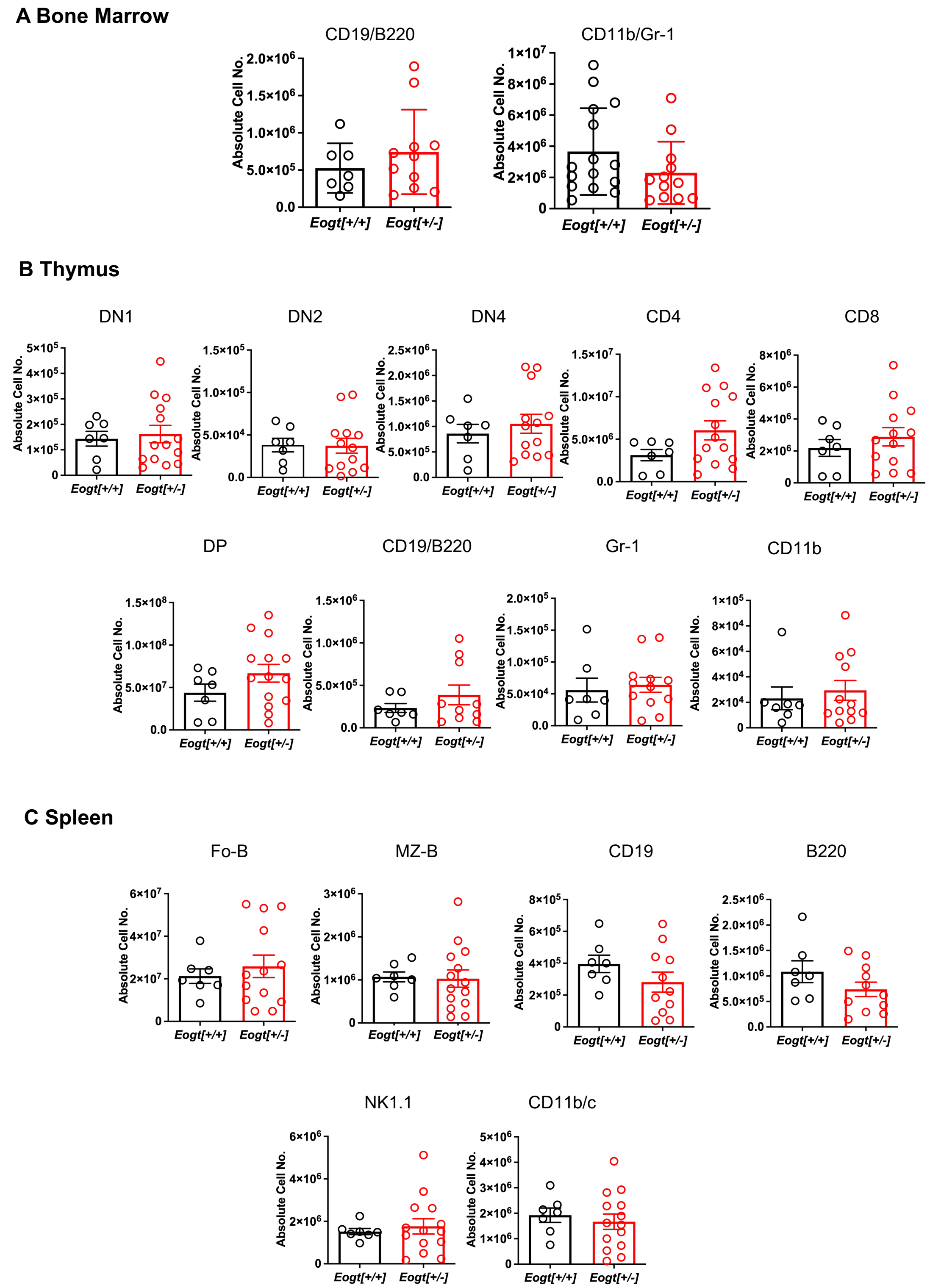

Supplementary Figure 4. Immune cell development in *Eogt* [+/+] and *Eogt*[+/-] mice. Related to Figure 2.

(A) Absolute cell numbers of CD19+ B220+ B cells, and granulocytes (CD11b+Gr-1+) in bone marrow. (B) Absolute cell numbers of CD44+CD25- (DN1), CD44+CD25+ (DN2), and CD44-CD25- (DN4) T cell progenitors, CD4+ SP, CD8+ SP, CD4+CD8+ DP T cells and B cells (B220+/CD19+), granulocytes (Gr-1+) and myeloid cells (CD11b+) in thymus. (C) Absolute numbers of follicular-B (Fo-B), marginal zone B (MZ-B), CD19+, B220+ B cells, natural killer T cells (NK1.1+), and dendritic cells (CD11b/c+). Each symbol represents a mouse of 7-8 weeks.

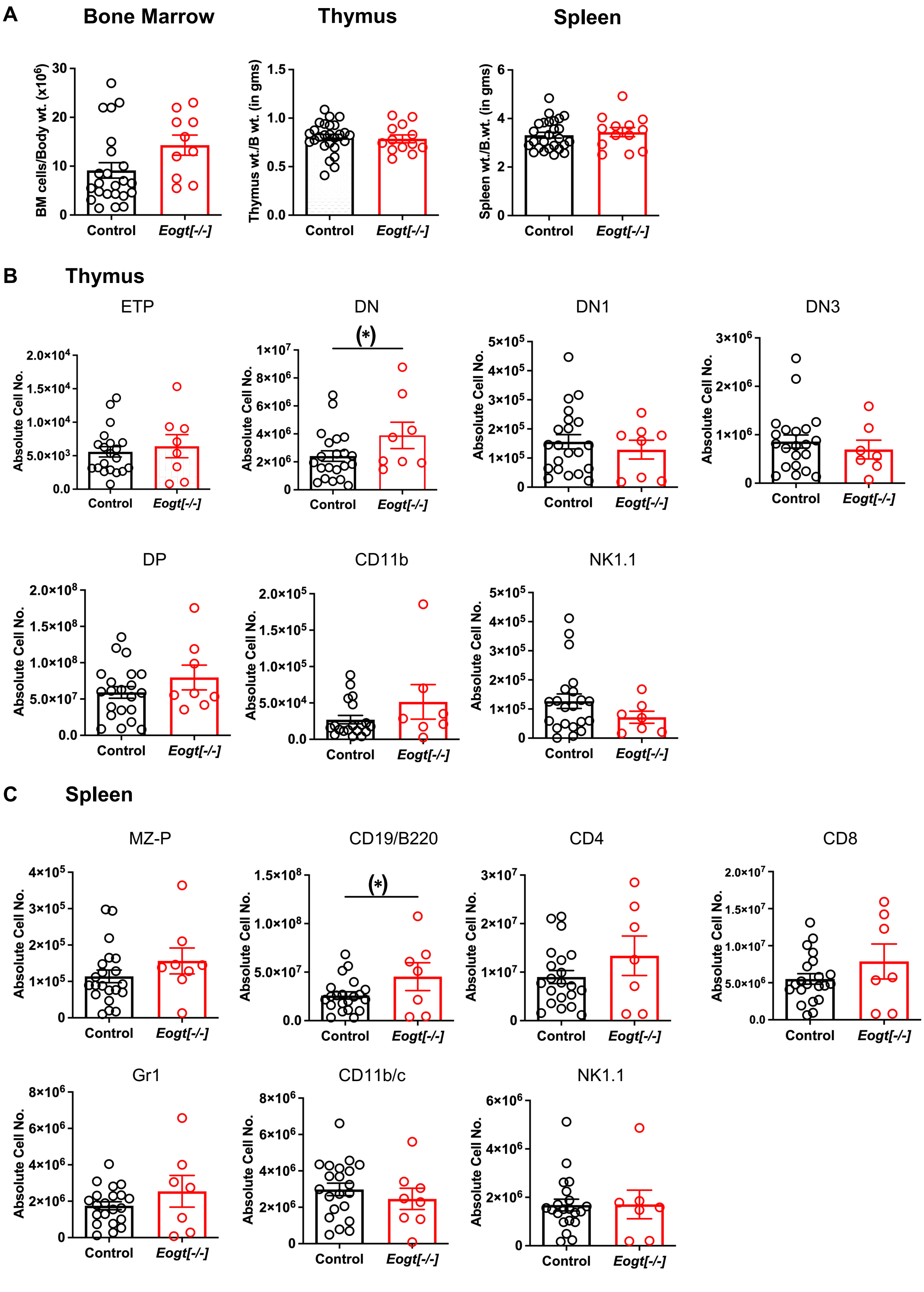

Supplementary Figure 5. Immune cell subsets in Control versus *Eogt*[-/-] mice. Related to Figure 2.

(A) Bone Marrow cellularity, thymus to body weight and spleen to body weight. (B) Absolute cell numbers of ETP, DN, DN1, DN3, DP, myeloid cells (CD11b+) and natural killer T cells (NK1.1+) in thymus. (C) Absolute cell numbers MZ-B, B cells (CD19+/B220+), CD4+, CD8+ T cells, granulocytes (Gr-1+), dendritic cells (CD11b/c), and natural killer T cells (NK1.1) in spleen. Each symbol represents a mouse of 7-8 weeks. Data presented as mean SEM. (*) $p < 0.05$ based on one-tailed Student t test.

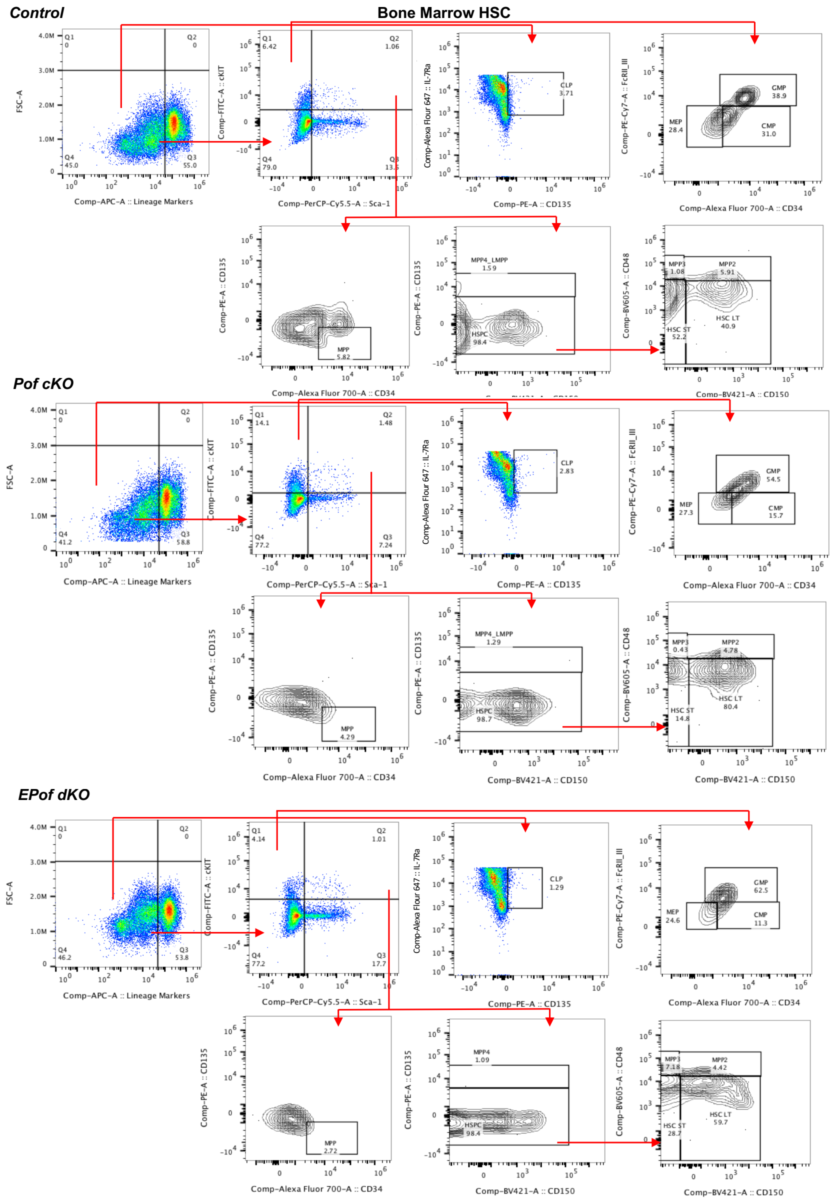

Supplementary Figure 6. Gating strategy for BM hematopoietic cell subsets. Related to Figure 5.

Flow cytometric profiles of targeted cell populations were selected after gating on live cells using Zombie NIR dye using the Cytek^{TM} Aurora flow cytometer from 7-8 weeks old, Control, Pof cKO and EPof dKO. HSPC populations were identified as follows:

LSK: Lin- cKit+ Sca1+,
Long-term hematopoietic stem cells (LT-HSC); Lin−Sca1+Kit+ CD150+CD135−,
Short-term hematopoietic stem cells (ST-HSC); Lin−Sca1+Kit+ CD150−CD135−,
Multipotent progenitors (MPP); Lin−Sca1+Kit+ CD150−CD135+,
Lymphoid primed multipotent progenitor cells (LMPP); Lin−Sca1+Kit+ CD135^{high},
Common lymphoid progenitors (CLP); Lin−KitintCD135+IL7Ra+,
Granulocyte/macrophage progenitors (GMP), Lin−Kit+Sca−FcRII/III+CD34+,
Megakaryocyte's erythrocytes progenitors' cells (MEP); Lin−IL-7R−c-kit+Sca-1−CD34−FcRII/III^{low}
Common myeloid progenitors (CMP); Lin−Kit+Sca−FcRII/III−CD34+,
Multipotent Progenitors cells 2 (MPP2); Lin−Kit+Sca+CD150+CD48+,
Multipotent Progenitors cells 3 (MPP3); Lin−Kit+Sca+CD150-CD48+
Multipotent Progenitors cells (MPP4); Lin−Kit+Sca+CD135+CD150-

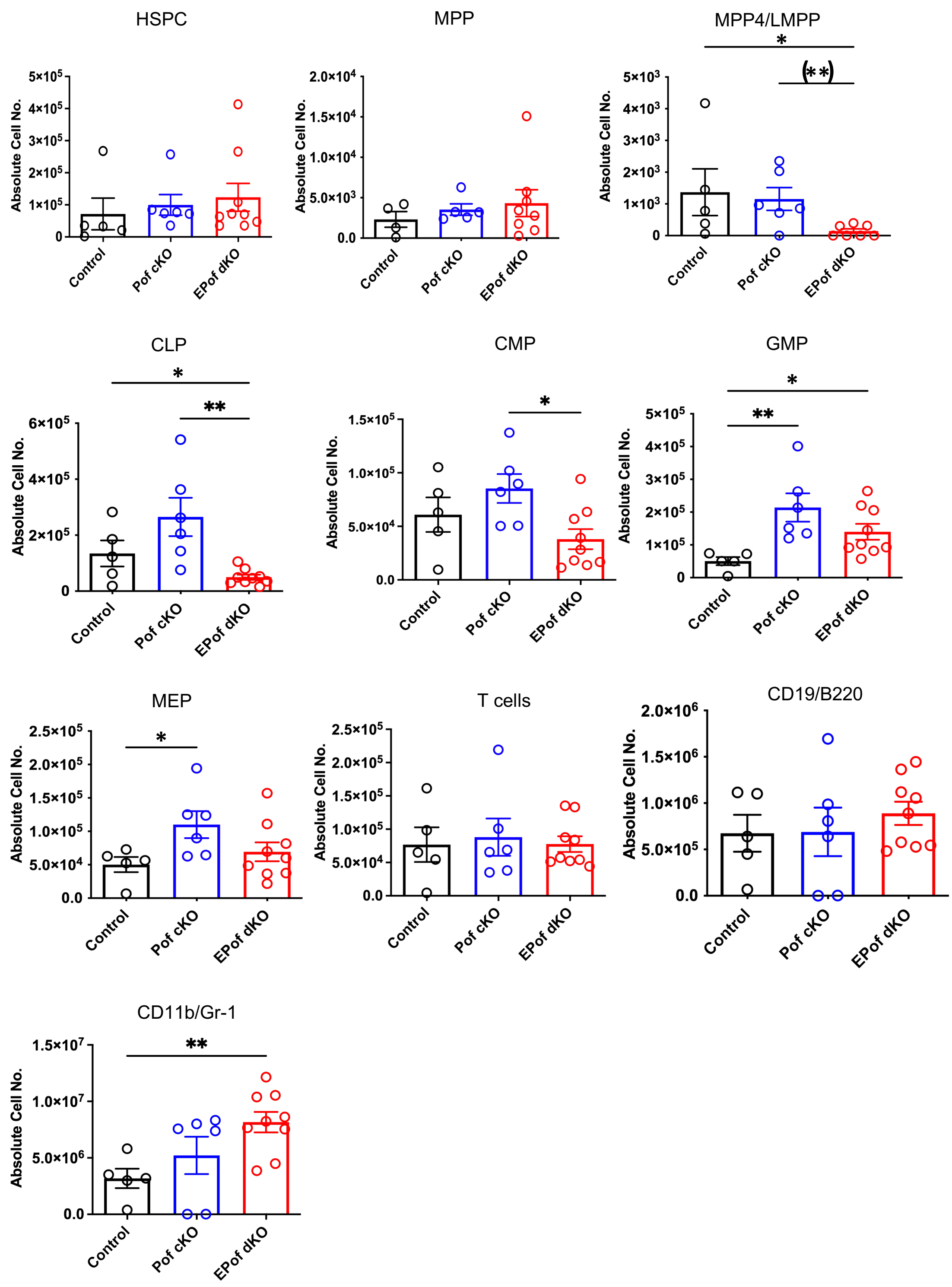

Supplementary Figure 7. Hematopoietic stem progenitors in Pof cKO and EPof dKO BM. Related to Figure 5.

Hematopoiesis, lymphoid and myeloid cell development were compared in Pof cKO versus EPof dKO versus Control mice with no *Vav1*-iCre. Each symbol represents a mouse of 7-8 weeks. Absolute cell numbers of different hematopoietic stem progenitor cells (HSPCs); multipotent progenitor (MPP) and multipotent progenitor-4 (MPP4)/LMPP cells, common lymphoid progenitor (CLP) common myeloid progenitor (CMP), granulocytes monocytes progenitor (GMP), megakaryocytes erythrocytes progenitors (MEP), T cells and B cells (B220/CD19) and granulocytes (CD11b/Gr-1) subsets.

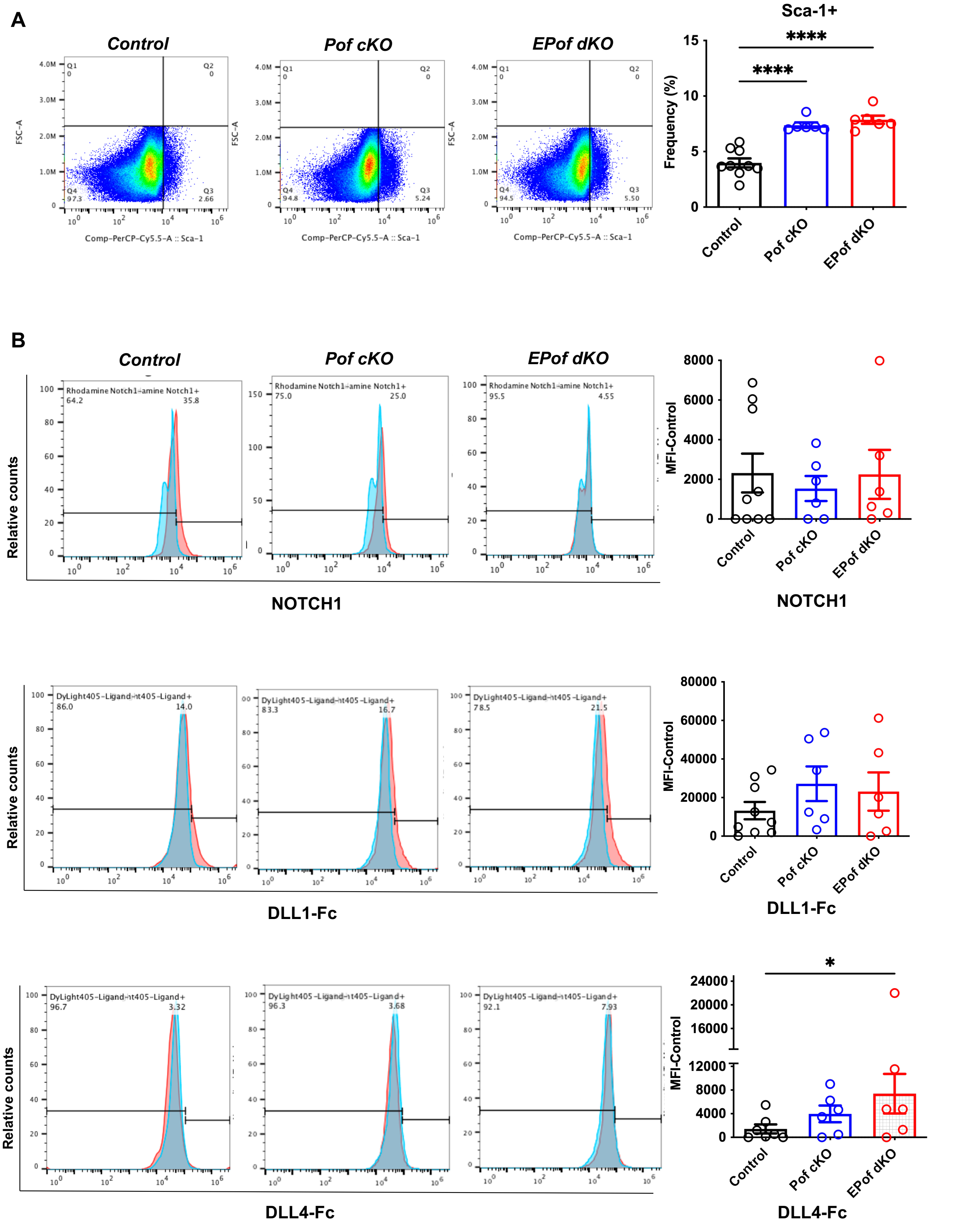

Supplementary Figure 8. Notch ligand binding to Lin-Sca1+ cells. Related to Figure 5.

Gating of Lin-Sca1+ cells and frequency (%) in triplicate samples of BM from Control (n=3), Pof cKO (n=2) and EPof dKO (n=2) 6-7 weeks old mice. All replicates are shown. Flow cytometry profiles of NOTCH1 cell surface expression, and binding of DLL1-Fc or DLL4-Fc to Lin-Sca1+ cells from the same mice performed in triplicate.

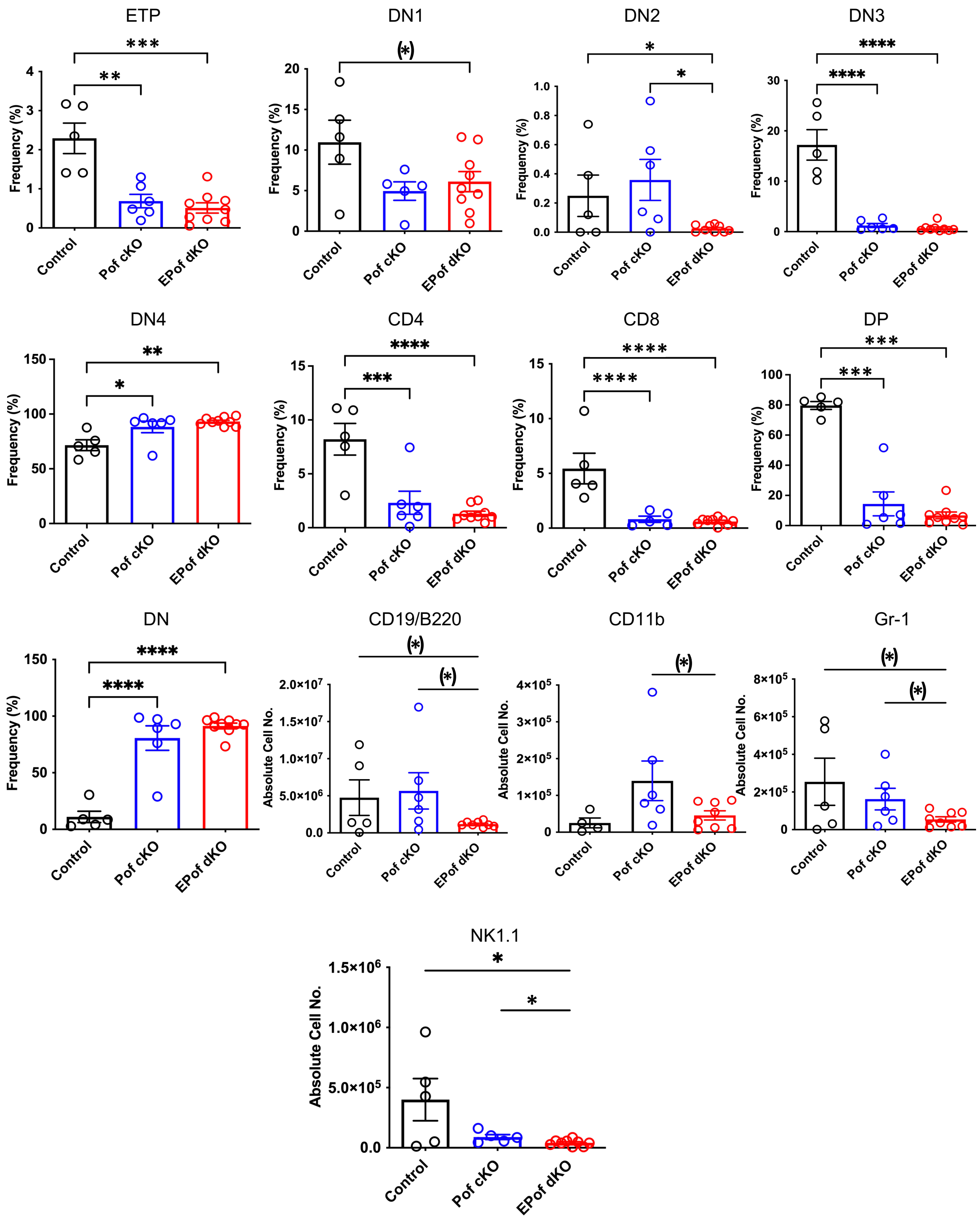

Supplementary Figure 9. Thymic immune cell subsets in Pof cKO and EPof dKO mice. Related to Figure 6.

Frequency(%) of different T-cell subsets; ETP(DN1 cells that were cKit/CD117+), and stages of different double negative (DN) T cells; CD44+CD25- (DN1), CD44+CD25+ (DN2), CD44-CD25+ (DN3), and CD44-CD25- (DN4) T cell progenitors, CD4, CD8, Double Positive (DP), Double negative (DN), and absolute numbers of B cells (B220/CD19), myeloid cells (CD11b), granulocytes (Gr-1) natural killer T-cells (NK1.1). Each symbol represents a mouse 7-8 weeks. Data are presented as mean SEM. *p <0.05, **p<0.01,***p<0.001, ,****p<0.0001 based on two-tailed Student t test and (*)p <0.05 based on one-tailed Student t test.

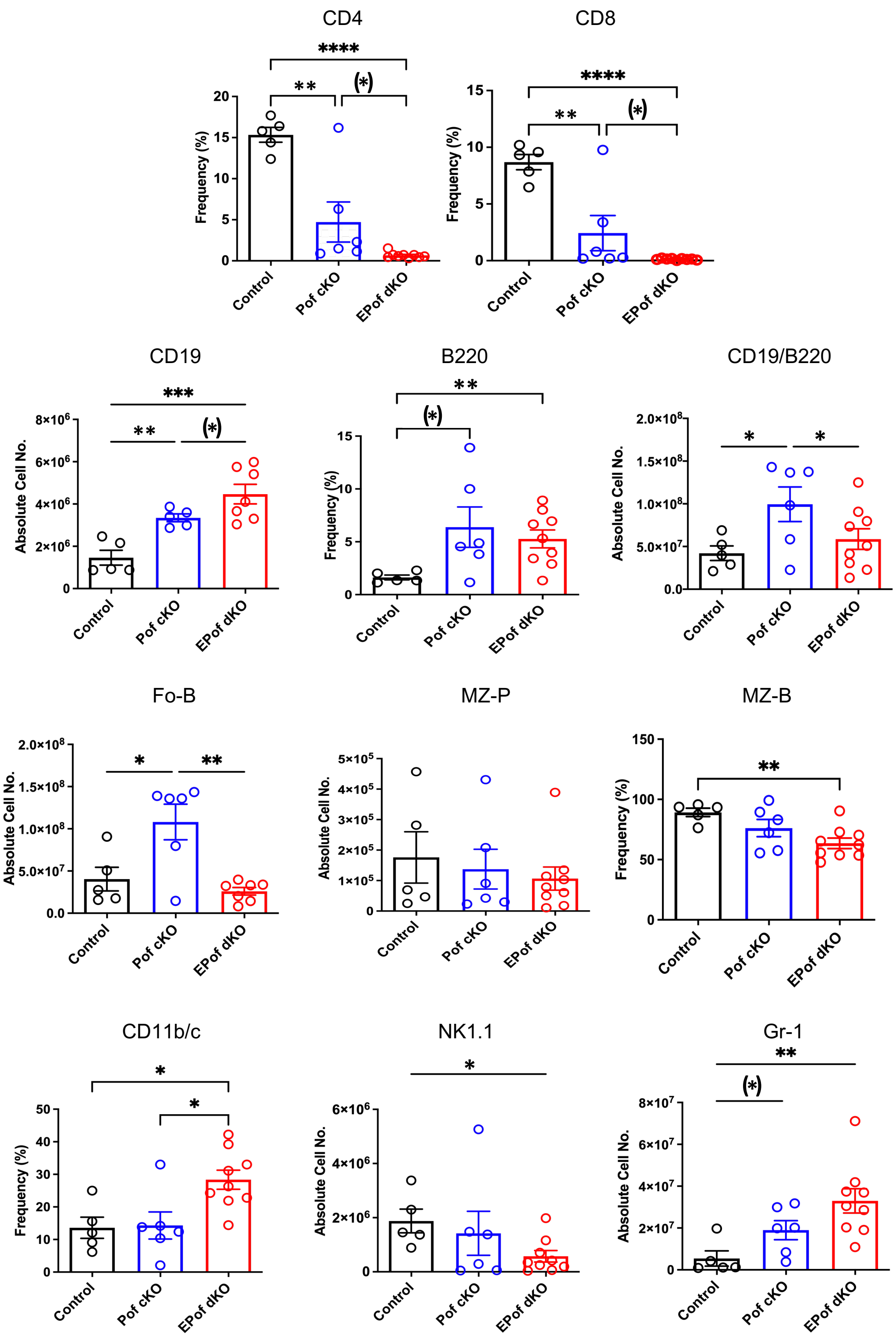

Supplementary Figure 10. Splenic immune cell subsets in Pof cKO and EPof dKO mice. Related to Figure 7.

Absolute cell numbers of CD19, B-cells (B220/CD19), follicular B cells (Fo-B), marginal zone precursors (MZ-P) cells, natural killer T cell (NK1.1) and granulocytes (Gr-1). Frequency (%) of splenic T-cell subsets; CD4, CD8, B220, marginal zone-B (MZ-B), dendritic cells (CD11b/c). Each symbol represents a mouse 7-8 weeks. Data are presented as mean SEM. *p <0.05, **p<0.01,***p<0.001, ,****p<0.0001 based on two-tailed Student t test and (*)p <0.05 based on one-tailed Student t test.

Supplementary Table 1. Primer sequences used in genotyping and qRT-PCR

Gene	Name	Sequence
Pofut1 F/del	PS644/For	*GGGTCACCTTCATGTACAAGTGAGTG*
	PS645/Rev	*ACCCACAGGCTGTGCAGTCTTTG*
Pofut1 F/wt	FB21/For	*CCAGGCTGATCACTTCTTGG*
	FB22/Rev	*CCCTGTCTCGAAAAAGCAAA*
Eogt	3rd loxF/For	*CCACCCGACCCCTGCCAGAACATAATGCTCTCTTGCATC*
	3rd loxR/Rev	*GCTGTCGCCAGAGGAGAGAGTGGGTGCTTACTTAC*
	25307 Rv/Rev	*CCAAGGCGGTCTTGGCCCAT*
Vav1-icre	FW internal/For	*CTAGGCCACAGAATTGAAAGATCT*
	RV Internal/Rev	*GTAGGTGGAAATTCTAGCATCATCC*
	FW Transgene/For	*AGATGCCAGGACATCAGGAACCTG*
	RV Transgene/Rev	*ATCAGCCACACCAGACACAGAGATC*
Hes1	For	*AAGGCAGACATTCTGGAAAT*
	Rev	*GTCACCTCGTTCATGCACTC*
Hes5	For	*GGACCAGAGGATGAGCTCGTT*
	Rev	*AGGAGGGAGCCTTCGGAAGA*
cMyc	For	*AGTGCTGCATGAGGAGACAC*
	Rev	*GGTTTGCCTCTTCTCCACAG*
Deltex1	For	*CATCAGTTCCGGCAAGAC*
	Rev	*ATGGTGATGCAGATGTCC*
CD25	For	*GGAATTGGTCTATATGCGTTGCTTA*
	Rev	*CATGTCTGTTGTGGTTTGTTGCTCT*
Tcf7	For	*TCCGAGTACATGGAGAAGCC*
	Rev	*GGGTAGGGCATGAGCAGATT*
Nrarp	For	*AGATACCGGATCCTCCGCTT*
	Rev	*CCGGTAATGGTTGTTTGGCG*
Gapdh	For	*AAGGTCATCCCAGAGCTGAA*
	Rev	*CTGCTTCACCACCTTCTTGA*
Hprt	For	*GGACCTCTCGAAGTGTTGGATAC*
	Rev	*GCTCATCTTAGGCTTTGTATTTGGCT*

Supplementary Table 2. Antibodies used in this work

Antibody	Fluorochrome	Isotype	Clone	Cat#	Company
CD4	PE	Rat IgG2a K	RM4.5	553048	BD Pharmingen
CD8a (ly2)	PE	Rat IgG2a K	53-6.7	553033	BD Pharmingen
CD45R/B220	PE-Cy7	Rat IgG2a, ϰ	RA3-6B2	103222	Biolegend
CD19	Alexa Flour 700	Rat IgG2a K	eBio1D3	56-0193-80	eBioscience
CD11b	FITC	Rat IgG2b K	M1/70	557396	BD Pharmingen
CD11c	FITC	Rat IgG2b K	N418	117306	Biolegend
Gr-1	APC	Rat IgG2b K	RB6-8C5	108412	Biolegend
CD3	APC	Rat IgG2b K	145-2C11	553066	BD Pharmingen
CD11b	APC	Rat IgG2b K	M1/70	101212	Biolegend
Ter 119	APC	Rat IgG2b K	TER-119	116212	Biolegend
CD19	APC	Rat IgG2b K	MB19-1	17-0191-81	eBioscience
IL-7Ra/CD127	Alexa Flour 647	Rat IgG2b K	A7R34	135011	Biolegend
CD117/cKit	FITC	Rat IgG2b K	2B8	105805	Biolegend
CD135/flk2	PE	Rat IgG2b K	A2F10	135305	Biolegend
CD16/32 or FCRIII/II	PE-Cy7	Rat IgG2b K	93	101317	Biolegend
CD34	Alexa Fluor 700	Rat IgG2b K	RAM35	56-0341-82	Invitrogen
Sca-1	PerCp-Cy5.5	Rat IgG2b K	D7	108123	Biolegend
CD150	BV421	Rat IgG2b K	TC15-12F12.2	115925	Biolegend
CD48	BV605	Rat IgG2b K	HM48-1	103441	Biolegend
CD4	FITC	Rat IgG2b K	RM4.5	11-0042-85	eBioscience
CD44	PE-Cy7	Rat IgG2b K	IM7	25-0441-81	eBioscience
CD25	PerCPCy5.5	Rat IgG2b K	PC61	102029	eBioscience
CD117 (cKit)	APC	Rat IgG2b K	1B8	553356	BD Pharmingen
CD19	PE-Cy7	Rat IgG2b K	6D5	115520	Biolegend
NK1.1	PerCPCy5.5	Rat IgG2b K	PK136	61-5941-80	eBioscience
IgM	APC	Rat IgG2b K	II/4I	17579082	eBioscience
CD21/35	PerCPCy5.5	Rat IgG2b K	7E9	123415	Biolegend
CD23	Alexa Flour 700	Rat IgG2b K	B3B4	101631	Biolegend
CD45.2	Pacific Blue	MOUSE SJL	104	109820	Biolegend
CD45.1	BV786	Mouse	A20	740889	BD Biosciences
CD8	PerCp-Cy5.5	Rat IgG2a K	53-6.7	553036	BD Pharmingen
AffiniPure F(ab9)2 Frag goat-anti-human IgG, Fcγ Frag Spec	APC	Goat-anti-human IgG		109-136-170	Jackson ImmunoResearch
AffiniPure F(ab9)2 Frag goat-anti-human IgG, Fcγ Frag Spec	DyLight 405	Goat-anti-human IgG		109-476-170	Jackson ImmunoResearch
Rhodamine Red-X-conjugated donkey anti-sheep IgG	Rhodamine Red-X	Donkey anti-sheep IgG		713-295-003	Jackson ImmunoResearch

CD16/ CD32 (mouse Fc block)	Rat anti-mouse	2.4G2	553142	BD Pharmingen
NOTCH1	Ag-purified polyclonal sheep	aa19-526	AF5267	R&D Systems
